# Correcting for selection bias after conditioning on a sum score in the Ising model

**DOI:** 10.3758/s13428-025-02820-1

**Published:** 2025-11-10

**Authors:** Jesse Boot, Jill de Ron, Jonas Haslbeck, Sacha Epskamp

**Affiliations:** 1 Department of Psychological Methods, University of Amsterdam, Amsterdam, The Netherlands; 2 Department of Clinical Psychological Science, Maastricht University, Maastricht, the Netherlands; 3 Department of Psychology, National University of Singapore, Singapore, Singapore

**Keywords:** Ising model, Selection bias, Sum score selection, Berkson’s bias

## Abstract

In psychological studies, it is common practice to select a sample based on the sum score of the modeled variables (e.g., based on symptom severity when investigating the associations between those same symptoms). However, this practice introduces bias if the sum score selection imperfectly defines the population of interest. Here, we propose a correction for this type of selection bias in the Ising model, a popular network model for binary data. Possible applications of our correction are when one wants to obtain (1) full population estimates when only the sum score subset of the data is available, and (2) improved estimates of a subpopulation, if we observe a mixture of populations that differ from each other in the sum score. In a simulation study, we verify that our correction recovers the network structure of the desired population after a sum score selection using both a node-wise regression and a multivariate estimation of the Ising model. In an example, we show how our correction can be used in practice using empirical data on symptoms of major depression from the National Comorbidity Study Replication (*N* = 9,282). We implemented our correction in four commonly used R packages for estimating the Ising model, namely *IsingFit*, *IsingSampler*, *psychonetrics,* and *bootnet.*

## Introduction

Network modeling has become increasingly popular in psychological research (Borsboom et al., 2021; Robinaugh et al., 2020; Contreras et al., 2019). In psychological network models, the nodes in the network represent variables, such as symptoms of a mental disorder or questionnaire items, and the edges represent the conditional associations between the variables (Epskamp & Fried, 2018). For binary variables, the Ising model (Ising, 1925) is the most popular network model (Finnemann et al., 2021). This model has now been applied to many psychological constructs, including depression (Cramer et al., 2016), attitudes (Dalege et al., 2016), substance abuse (Rhemtulla et al., 2016), and intelligence (Savi et al., 2019).

In many network analysis studies, the Ising model is estimated based on a subset of the data that is defined with respect to a minimum sum score of the modeled variables (e.g., Hakulinen et al., 2020; Heeren & McNally, 2018; Van Borkulo et al., 2015; Van Rooijen et al., 2018). This will change the resulting network model compared to the network model of the full sample. For example, if all edges represent positive linear associations in the full data, then subsetting the data using only cases above a certain sum score leads to an attenuation of positive associations, and absent or weak associations can become negative (De Ron et al., 2021; Epskamp et al., 2022). If a researcher is really only interested in the conditional associations of the sample selected by a sum score, this change in edges is not a problem. The conditional associations are valid for the specific sample. However, the edges are biased if a researcher’s target of inference is not identical to the population selected by a sum score—for example, a population defined by a latent trait (Haslbeck et al., 2021b).

Moreover, the change in edges is problematic if a researcher wants to substantively interpret the associations, for example by using them to generate hypotheses about causal effects between the variables. Even if we analyze exactly the probability distribution of interest, hypotheses about causal effects are invalid after conditioning on a sum score (Griffith et al., 2020; Haslbeck et al., 2021b). This is because the attenuation of edges after a sum score selection does not reflect a causal relationship between the variables but are the result of conditioning on a common effect, i.e., collider, of the variables, namely their sum score.^1^ Meanwhile, generating hypotheses about causal effects is arguably a central motivation of researchers to estimate network models (Borsboom & Cramer, 2013; Epskamp et al., 2018a).

**Fig. 1:**
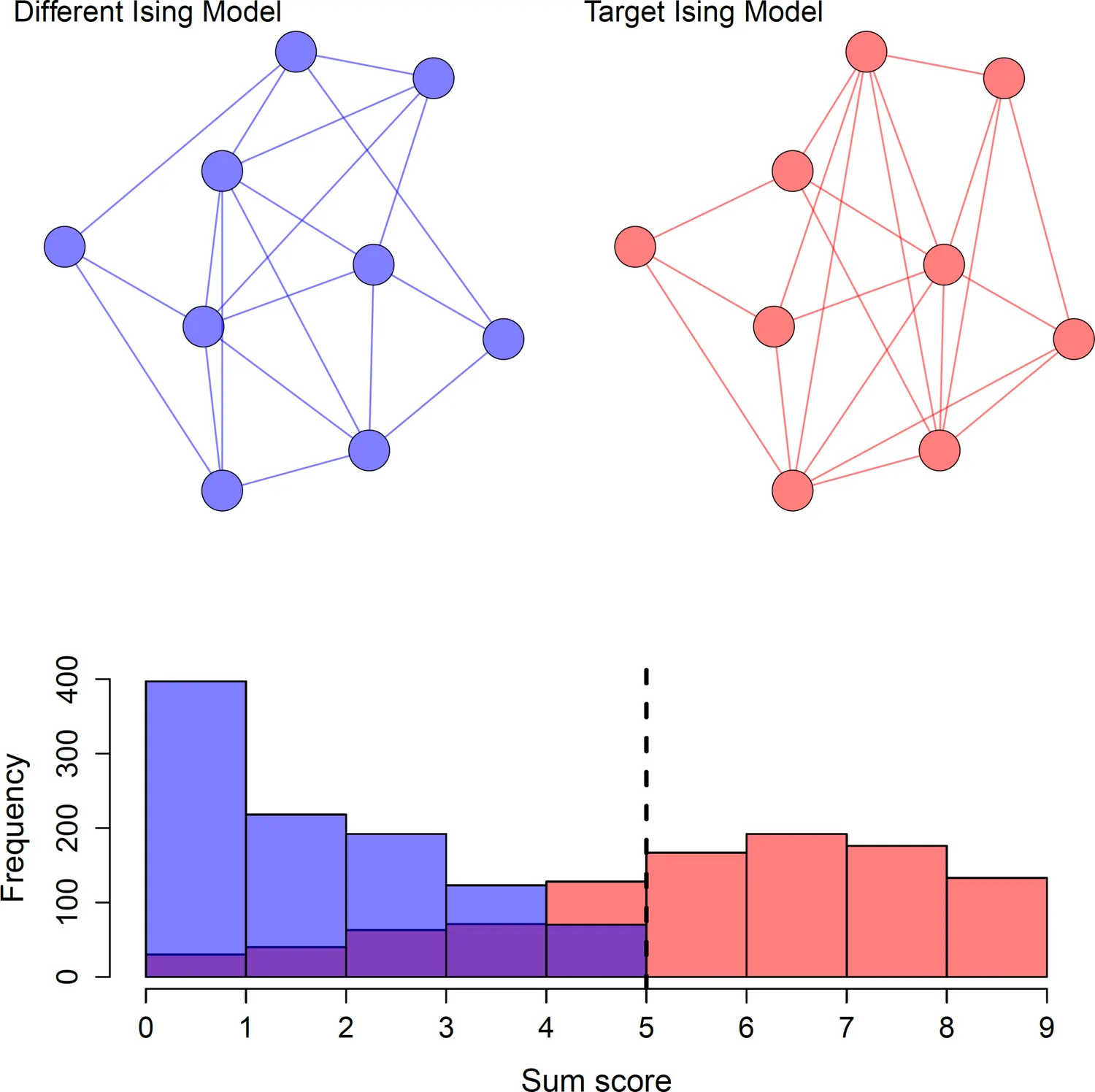
Illustration of when our correction would apply. *Note*. There are two Ising models that generated the data. The red “target Ising model” is the model that the researcher is interested in. A sum score cutoff criterion of 5 as indicated by the black dotted line only selects data from the target population, completely excluding the different blue population, but also excludes some people from the target population, as the purple part of the histogram is where the populations overlap

A solution to bias after a sum score selection would be to simply not condition on a sum score (Dablander et al., 2019; Haslbeck et al., 2021b).^2^ However, we see two scenarios, both illustrated in Fig. 1, in which this is unsatisfactory and in which we would prefer to subset on the sum score and correct estimates afterwards to get to improved estimates of the Ising model in the population of interest. The two scenarios are as follows:

1. There is only data available on part of the population that is selected by a sum score (Fig. 1, the red target population that reaches the chosen sum score cutoff), but the population of interest is the target population regardless of the sum score (Fig. 1, the full red target population, including cases in the purple part of the distribution).
2. The sample consists of two or more subpopulations: a target population (Fig. 1, the red target population) and at least one different population (Fig. 1, the blue different population) that partly overlaps with the target population (the purple part of the figure represents overlap between the populations). A sum score cutoff criterion selects part of the target population, but not everyone. Moreover, no one in the different population reaches the chosen cutoff criterion. In Fig. 1 it can be seen that the purple part of the distribution does not reach the sum score that is at the black dotted line but consists partly of people from both populations.

Scenario 1 could arise, for example, if a study had a diagnosis of a mental disorder as inclusion criterion, which is based on the sum score of symptoms, as the definition of mental disorders heavily depends on the presence of symptoms (Borsboom et al., 2016). Scenario 2 could arise when one assumes the presence of two or more subpopulations that are captured by different Ising models and that are not captured by any measured variable that could be used to distinguish the subpopulations (i.e., it is latent).^3^ While we cannot verify whether this is indeed the case, it may be plausible in situations in which two assumed populations closely align with the sum score. Again, an example is the case of a mental disorder, where one could assume that there is a latent trait, like vulnerability, which is closely related but not equivalent to the sum score of symptoms.

In the two scenarios described above, estimating an Ising model based on the subset data leads to biased estimates for the target population at hand. In this paper, we propose a correction to estimate the Ising model for the target population from data that is selected by a sum score cutoff criterion. This correction leads to unbiased estimates of the Ising model for the target population in the scenarios described above. We implemented our correction in four frequently used packages to estimate the Ising model: *IsingFit* (Van Borkulo & Epskamp, 2023), *IsingSampler* (Epskamp, 2023a), *bootnet* (Epskamp et al., 2018a, 2018b), and *psychonetrics* (Epskamp, 2023a, 2023b).

We will first describe how our proposed estimation method corrects for selection bias in the Ising model stemming from selecting with the sum score of the modeled variables. Second, we demonstrate that our correction works in a simulation study using empirical network parameters. Because the network of the full population is known in this situation, we can evaluate how well our correction recovers the data-generating network, compared to estimation as usual. Finally, we give an example using simulated data and the National Comorbidity Study Replication data (NCR-R; Kessler & Merikangas, 2004) to guide researchers on the inferences that they can make after using our correction with empirical data.

## Correcting selection bias

The most popular network model for binary data is the Ising model (Ising, 1925; Epskamp, 2017), which models the probability distribution over multivariate binary data with a set of thresholds for each variable and pairwise associations between each pair of variables. The Ising model can be estimated using multivariate and univariate estimation. Multivariate estimation optimizes the model parameters given the available data, using the joint probability distribution to estimate all parameters simultaneously (e.g., Epskamp et al., 2022). Univariate estimation maximizes the pseudo-likelihood of the data by fitting multiple logistic regressions, where the coefficients correspond directly to the conditional associations of the Ising model (Epskamp et al., 2022; Marsman et al., 2018). Although multivariate estimation should generally be more accurate (e.g., Brusco et al., 2022; or for the case of the exponential random graph model, see Van Duijn et al., 2009), it is computationally intensive. Though the Ising model remains tractable for most network sizes encountered in psychological network research, the R implementations commonly used for these networks often cannot estimate networks with more than 20 nodes (Finnemann et al., 2021; Epskamp, 2017; Van Borkulo et al., 2014).

### Multivariate estimation correction

The Ising model can be expressed as a joint probability distribution as follows:

$$Pr\left({{\boldsymbol{Y}}}_{{\boldsymbol{p}}}={{\boldsymbol{y}}}_{{\boldsymbol{p}}}\right)=\frac{1}{Z}exp\left(\sum\limits_{i}{{\tau }_{i}y}_{pi }+\sum\limits_{< i,j>}{{\omega }_{ij}y}_{pi}{y}_{pj}\right)$$

where ${{\boldsymbol{Y}}}_{{\boldsymbol{p}}}$ is a vector of binary responses of person p on m items or nodes and ${{\boldsymbol{y}}}_{{\boldsymbol{p}}}$ is a specific state of ${{\boldsymbol{Y}}}_{{\boldsymbol{p}}}$ (e.g., a vector of 0’s and 1’s when a symptom is absent or present, respectively).^4^Z is a normalizing constant with respect to the data (Epskamp et al., 2022) such that the probability of all possible realizations of ${{\boldsymbol{Y}}}_{{\boldsymbol{p}}}$ sum up to 1:

$$Z = \sum_{{\boldsymbol{y}}}exp\left(\sum\limits_{i}{{\tau }_{i}y}_{pi}+\sum\limits_{< i,j>}{{\omega }_{ij}y}_{pi}{y}_{pj}\right)$$

The part of the equation that is being summed here is called the potential, Pot(${{\boldsymbol{Y}}}_{{\boldsymbol{p}}}={{\boldsymbol{y}}}_{{\boldsymbol{p}}}$), which can be understood as the unnormalized probability that ${{\boldsymbol{Y}}}_{{\boldsymbol{p}}}$ shows response pattern ${{\boldsymbol{y}}}_{{\boldsymbol{p}}}$. In the potential function of the Ising model ${\tau }_{i}$ represents the threshold of node i, which can be understood as a preference of the node for a specific value. Moreover, ${\omega }_{ij}$ represents the pairwise interaction between nodes i and j. If this parameter is positive, states where nodes i and j have the same value become more likely. Conversely, if ${\omega }_{ij}$ is negative, states where nodes i and j have different values become more likely.

Critically for our correction, when estimating the Ising model after a sum score selection, bias results from the fact that Z still sums over all possible realizations of ${{\boldsymbol{y}}}_{{\boldsymbol{p}}}$. However, we know with certainty that response patterns where the sum score (${s}_{p}$) does not reach our cutoff criterion k are no longer possible, because for all cases in the data it holds that

$${s}_{p}=\sum\limits_{i}{y}_{pi}\ge k.$$

To correct this, we have to instead model the conditional probability distribution:

$$Pr\left({{\boldsymbol{Y}}}_{{\boldsymbol{p}}}={{\boldsymbol{y}}}_{{\boldsymbol{p}}}| {s}_{p}\ge k\right)$$

We can do this by summing only over the response patterns where ${s}_{p}\ge k$ when calculating Z:

$${Z}^{\left(k\right)}=\sum\limits_{y, s\ge k}exp\left(\sum\nolimits_{i}{\tau }_{i}{y}_{pi}+\sum\limits_{< i,j>}{{\omega }_{ij}y}_{i}{y}_{j}\right)$$

If we use ${Z}^{\left(k\right)}$ instead of Z in the original Ising model, the model should be estimated without bias resulting from the sum score selection. For example, in the very simple case of three variables that are either present (1) or absent (0) and a sum score cutoff criterion of 2, this would mean that we calculate Z by summing the potential of all possible response patterns that are still possible after our selection:

$$Z = Pot({\boldsymbol{Y}} = [\mathrm{1,1},0]) + Pot({\boldsymbol{Y}} = [\mathrm{1,0},1]) + Pot({\boldsymbol{Y}} = [\mathrm{0,1},1] + Pot({\boldsymbol{Y}} = [\mathrm{1,1},1]).$$

We simply excluded all other response pattern potentials and can then continue to estimate the Ising model parameters as usual. We note that our correction makes multivariate estimation much more tractable, by reducing the space over which the normalizing constant is calculated.

### Univariate estimation correction

The intuition behind our correction for univariate estimation is similar as in multivariate estimation. Instead of computing the pseudo-likelihood of the data, in which nodewise regressions are used to model each variable once as dependent variable and all other variables as dependent variables, we compute pseudo-likelihood of the data conditional on our cutoff criterion k. That is, the logistic regression model for the first variable, ${y}_{1}$, given all the other variables ${\mathbf{y}}_{p}^{\left(-1\right)}$ and the condition that the sum score reaches our cutoff criterion k, becomes:

$$Pr({Y}_{1} ={y}_{1}| {{\boldsymbol{Y}}}_{p}^{\left(-1\right)}={\mathbf{y}}_{p}^{\left(-1\right)}, {s}_{p}\ge k) = \frac{Pr({Y}_{1} ={y}_{1}, {{\boldsymbol{Y}}}_{p}^{\left(-1\right)}={\mathbf{y}}_{p}^{\left(-1\right)},{s}_{p}\ge k)}{\sum_{{y}_{i}}Pr({Y}_{1} = {y}_{i},{{\boldsymbol{Y}}}_{p}^{\left(-1\right)}={\mathbf{y}}_{p}^{\left(-1\right)}, {s}_{p}\ge k )}$$

in which $\sum_{{y}_{i}}$ takes the sum over both possible outcomes of ${y}_{i}$. However, there is one situation in which we do not have to sum over both possible outcomes of ${y}_{i}$: when the sum of the other variables equals k-1, as the response ${y}_{1}$ can only be 1 to have a sufficient sum score to be included in the sample.

Let us illustrate this with an example Ising model with five symptoms, ${y}_{1}$ to ${y}_{5}$, in which 1 indicates that a symptom is present, and 0 that it is absent. We compute the sum score for everyone (${s}_{p}$), and we only keep individuals in our data analysis who reach at least cutoff criterion k (i.e., ${s}_{p}$ ≥ k). If we now fit a logistic regression of ${y}_{1}$ with all other symptoms as predictors, there is one situation in which our cutoff criterion gives information about the outcome, namely when the sum score of the predictors is equal to k-1. This is because we already know with certainty that

$$Pr\left({y}_{1}=1 |{s}_{p}^{(-1)}=k-1, {s}_{p}\ge k\right)=1,$$

where ${s}_{p}^{(-1)}$ is the sum score without ${y}_{1}$ included, ${\sum }_{i=2}^{p}{y}_{i}$. If it is known that if the sum of the other symptoms is equal to k-1, the predicted symptom must be present. Thus, to correct for selection bias, we have to set the likelihood of the data to 1 for all cases where this is true. In practice, this means that in the univariate regression models, cases for which the predictors sum to *k* - 1 can be removed.

## Simulation study

The main goal of our simulation study was to compare the performance of our correction with uncorrected, regular estimation in recovering the “true” network structure after selection based on a sum score criterion. We used a true network structure to generate binary data from, which we consider to be plausible in psychological network studies (i.e., a dense network with many positive edges).

### Methods

#### Simulation procedure

We used the statistical software R for all analyses (R Core Team, 2016). The R code and materials are available at https://github.com/Jesse291847/Correcting-for-selection-bias. The simulation study consisted of three steps. In step 1, we constructed a “true” network from which we generated binary data. In step 2, we selected cases from the generated data that reached the chosen sum score criterion, and we estimated networks with and without the correction, with three different packages, namely *IsingFit*, *IsingSampler*, and *psychonetrics*. In step 3, we compared how much the estimated network with and without correction resembled the true network.

We estimated networks with four different sample sizes (i.e., 500, 1,000, 2,500, and 5,000) and three different sum score selection criteria (i.e., 0, 2, and 5). In this context, the sample size refers to the sample size after a sum score selection, if applicable. We chose a cutoff criterion of zero to have a baseline accuracy of each estimator to compare the performance of our correction to. We chose 2 and 5 to investigate how detrimental a higher sum score cutoff criterion is for the performance of recovering the true network. We ran each condition 100 times and estimated six networks in each run, i.e., a corrected and an uncorrected network with all three packages. The only exception was when estimating on the full sample, where we estimated three networks, since in this case there is no difference between the corrected and the uncorrected network. This resulted in a total of 1,200 runs (4 sample sizes × 3 cut-off criteria × 100 repetitions). We describe each step of the simulation in more detail in the following sections.

#### Step 1: Data generation

We used the edge weight and threshold parameters from Cramer et al. (2016) to simulate data using the *IsingSampler* function (Epskamp, 2023a). In principle, any network structure can verify that our correction works. However, using empirically informed parameters ensures that our true network has structure that could be plausible in empirical research. The network from Cramer et al. (2016) was estimated using data from the first interview of the Virginia Adult Twin Study of Psychiatric and Substance Use Disorders (VATSPSUD; Kendler & Prescott, 2006; Prescott et al., 2000). These data contain information on the presence or absence of 14 disaggregated symptoms of depression from the Diagnostic and Statistical Manual of Mental Disorders, Third Edition, Revised (DSM-III-R; American Psychiatric Association, 1987) for 8,937 participants. Interviews were conducted by trained mental health professionals, who rated the presence of each symptom. When a symptom was present, the interviewer made sure the symptom was not caused by a physical problem.

**Fig. 2:**
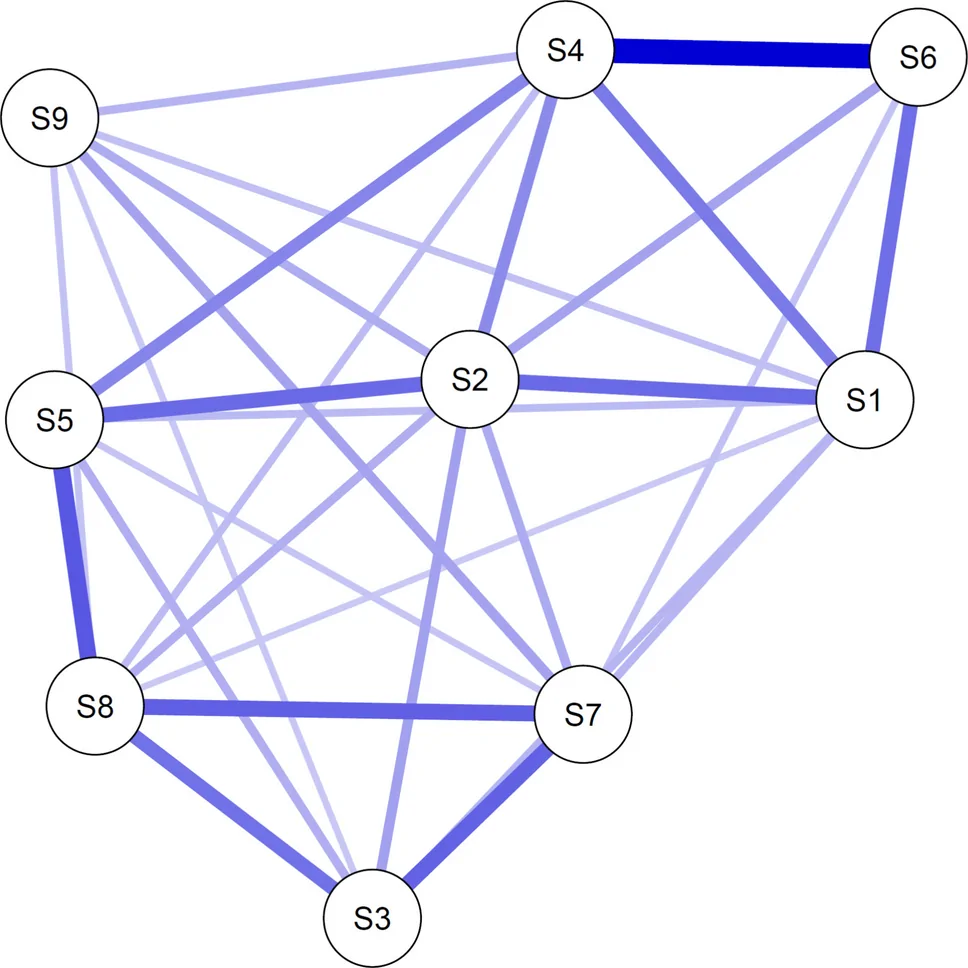
The true, data-generating network. *Note:* This network is based on the empirical weight parameters estimated by Cramer et al. (2016). Each node represents a depression symptom, and each edge represents a conditional association between two symptoms. The stronger the conditional association, the more saturated and thicker the edge is. Blue edges indicate positive pairwise associations. As we have changed the parameters, we do not show the names of the symptoms to emphasize that this is not an empirical network structure

We aggregated some of the symptoms so that the nine major depressive disorder (MDD) symptoms of the DSM-5 remained (American Psychiatric Association, 2013). For details on how we obtained the true network, we refer to Appendix A. Figure 2 shows the resulting true network. Even though we made slight adaptations to the original network, the resulting structure remains similar to structures expected in psychological empirical research. The density (i.e., the proportion of present edges of all possible edges) was .81; all of the 29 edges present were positive, and the average edge strength was .61.

#### Step 2: Network estimation

From the generated data, we selected cases that reached the sum score cutoff criterion (i.e., 0, 2, or 5). We used the subset of the data to estimate a corrected and an uncorrected network using three different packages: *IsingFit* (univariate estimation; Van Borkulo & Epskamp, 2023), *IsingSampler* (univariate estimation; Epskamp, 2023a), and *psychonetrics* (multivariate estimation; Epskamp, 2023a, 2023b). With the *IsingFit* function, we used node-wise logistic regressions combined with Lasso regularization (Tibshirani, 1996), with the extended Bayesian information criterion (EBIC) (Chen & Chen, 2008) model selection (where some parameters are forced to zero, and different models are compared to minimize the EBIC statistic; see Van Borkulo et al., 2014, for details). With the *IsingSampler* function, we used node-wise regressions with thresholding according to the AND rule (α=0.01), where only edges that are significant in both directions are included in the network. With the *psychometrics* package we used pruning, where the algorithm removes edges that are not significant (α=0.01) and then re-estimates the network.

#### Step 3: Outcome measures

We compared the estimated networks with the true network based on the total edge weight error, which is the difference between the true and the estimated edge weights. To compute the total edge weight error, we summed the absolute difference between the edge weights in the true network and the estimated network. In general, a lower total edge weight error indicates a more accurate estimation. Second, we used the outcome measures sensitivity (i.e., the proportion of true edges present correctly estimated) and specificity (i.e., the proportion of true absent edges correctly estimated to be zero) to assess the performance of model selection (in the case of eLasso estimation) and significance thresholding (in the other estimators). In general, higher sensitivity indicates more power to detect true edges, and high specificity indicates a low false positive rate. Ideally, sensitivity increases with sample size, and specificity is high regardless of sample size and fixed at (1-α) when using significance thresholding. Finally, as bias in our simulation setup will generally be towards edges being negative (e.g., De Ron et al., 2021), we looked at the proportion of spurious negative edges. This measure is complementary to the sensitivity measure. If a negative edge is estimated where there is a positive edge in the true network, the sensitivity will still increase (as sensitivity is agnostic to edge sign), but it will also increase the proportion of spurious negative edges.

### Results

**Fig. 3:**
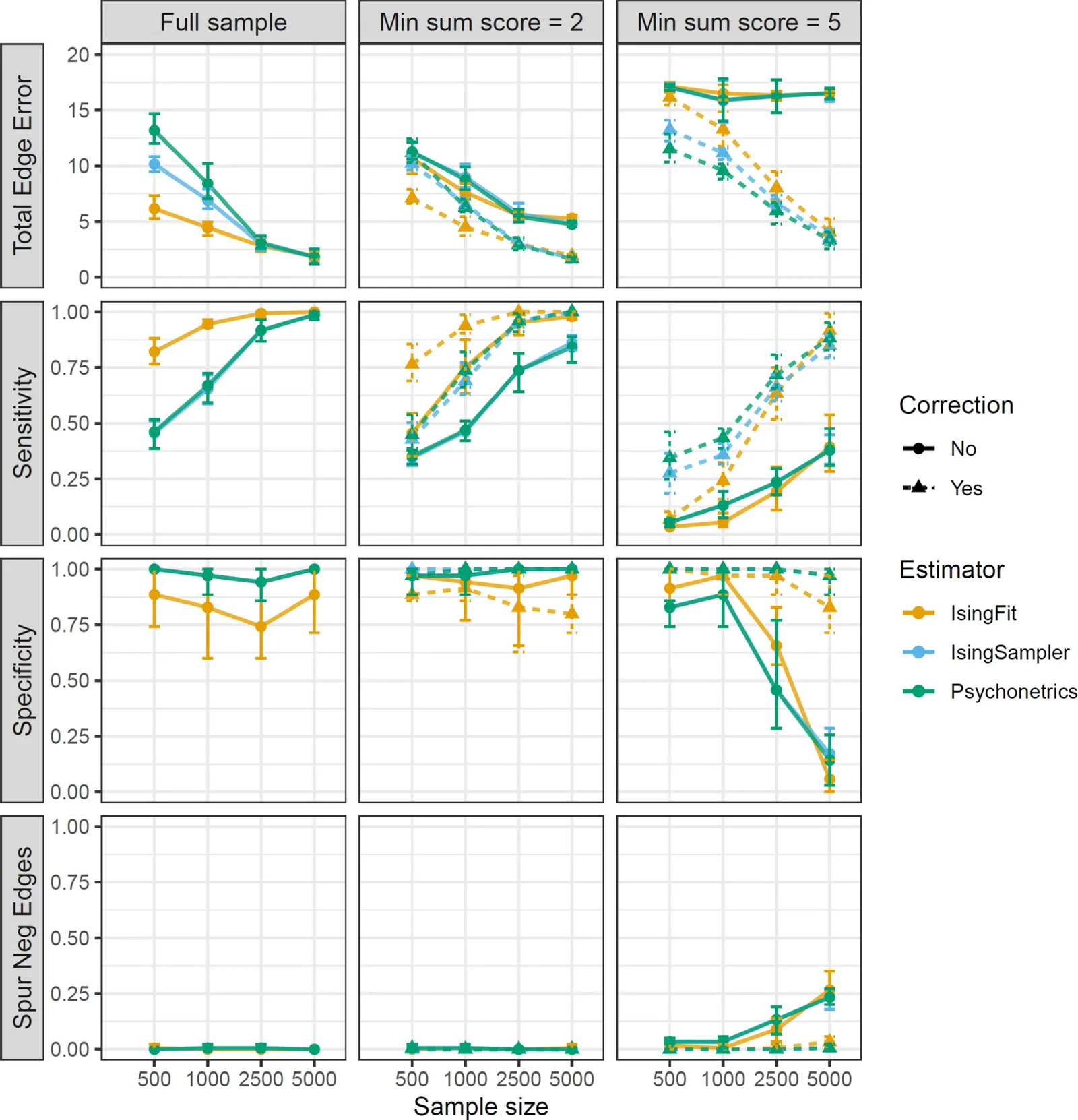
Mean results of outcome measures for 100 runs of simulation for different estimators. *Note.* The mean result of 100 simulations is shown on the *y*-axis, and the sample sizes of the simulated data on the *x*-axis. The different horizontal panels indicate our outcome measures—total edge weight error, sensitivity, specificity, and the proportion of spurious negative edges—while the different vertical panels indicate the different sum score selection criteria (0, 2, 5). Dashed lines are used for the corrected networks, whereas solid lines are used for the uncorrected networks. The colors of the lines correspond to the package used for estimation. Note that *IsingSampler* (node-wise estimation) and *psychonetrics* (multivariate estimation) performed similarly, and due to overlap they are not always both visible

Figure 3 shows the simulation results of 100 runs with different sample sizes and sum score cutoff criteria. Appendix B (Fig. 6) shows that results for total edge weight error are similar when we do not use any model selection. In each condition where we used a sum score criterion, the corrected networks show lower total edge weight error than the uncorrected networks. When the sum score criterion is high (i.e., ≥ 5), the difference between the corrected and uncorrected networks increases with sample size: the networks estimated with the correction improve drastically as the sample size increases, whereas the total edge weight error remains high for networks estimated without correction. This suggests that total edge weight error is mainly due to sampling variation for the corrected networks and mainly due to bias for the uncorrected networks.

Sensitivity and specificity are higher for the corrected networks than for the uncorrected networks, and this difference increases with sample size and sum score criterion. With increasing sample size, the corrected networks show a strong increase in sensitivity, whereas the uncorrected networks show only a small increase in sensitivity at higher sample sizes. Moreover, for a sum score criterion of 5, this increase for the uncorrected networks is likely partly due to the inclusion of spurious negative edges, since the proportion of spurious negative edges shows a similar increase as the sensitivity for the uncorrected networks. Specificity decreases with sample size, especially for the sum score criterion of 5 and the uncorrected networks. For the corrected networks, specificity remains close to 1 for all sample sizes. An exception is *IsingFit*, where specificity is consistently below 1 even when estimating a network based on the full sample. The low mean specificity is likely due to the fact that there are only seven edges missing in the true network, so that estimating one or two of those edges already leads to a large drop in specificity. Contrary to the other packages, *IsingFit* does not use α of 0.01, which could explain why this package detects more false positive edges.

## Empirical example

In this example we show that we can use our correction as a way to test the hypothesis that there are at least two latent populations that are closely related to the sum score (scenario 2, as described in the introduction). First, we show with simulated data how our correction can reject this hypothesis. Next, we show an example with empirical data that seems consistent with this hypothesis. However, we caution against interpreting findings from this type of analysis as evidence for the presence of latent populations that are related to the sum score. This is because other models could have produced similar results. For example, a homogeneous Ising model in which the parameters are moderated by the sum score could lead to the same results in network analysis as the presence of two different latent populations.

We focus on scenario 2, because we expect that the most prominent reason why researchers use sum score selection is because they suspect the presence of different latent populations, for example, a population with a resilient network structure and a population with a vulnerable network structure (Borsboom & Cramer, 2013). Since these latent populations cannot be observed directly, we could use the sum score as a way to try to exclude people with a resilient network from the analysis, assuming that individuals with a resilient network will have a very low probability to reach a high sum score. Therefore, we can use the sum score as a proxy for identifying the latent populations.

### A simulated case

**Fig. 4:**
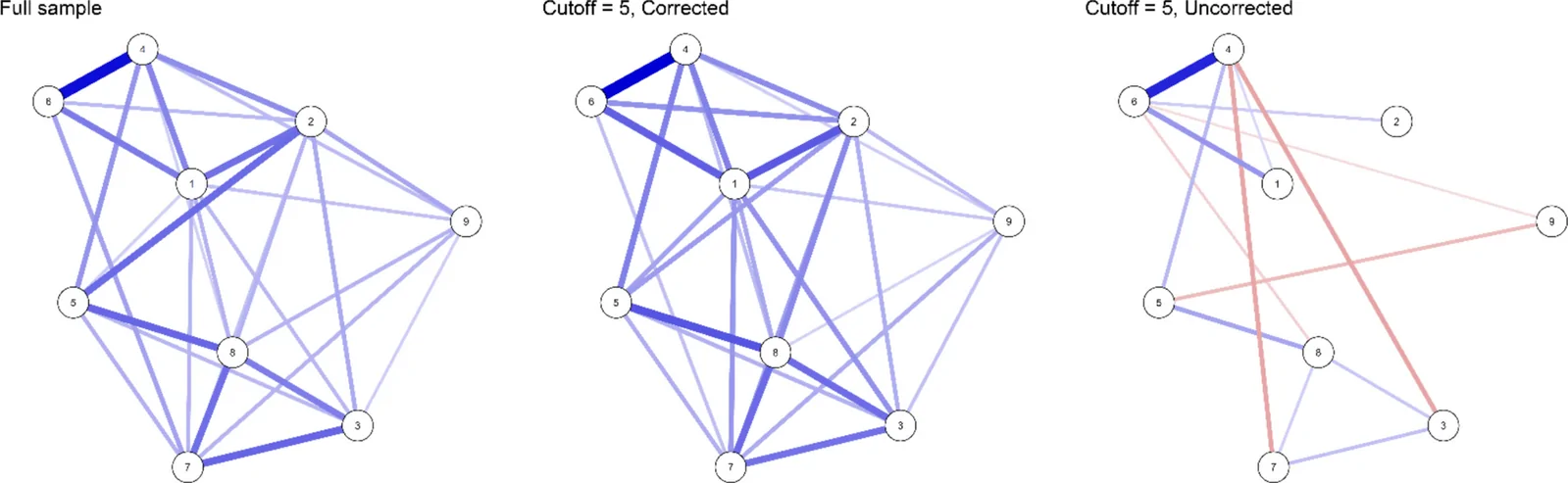
Three networks estimated from simulated data. *Note.* Three networks estimated from data simulated with the parameters shown in Appendix A (Table 1). All networks are estimated with a sample size of 5,000. The network on the left is estimated on a sample without restrictions on the minimum sum score, while the networks in the middle and on the right are estimated on data where the sum score of the variables was at least 5. The middle network shows networks estimated with our correction and the right network without our correction. All networks were estimated with the *IsingSampler* package (α=0.05 with the AND rule)

We used the same network as in our simulation study (Fig. 3) to generate two datasets with a sample size of 5,000. The first dataset had no restrictions on the sum score, and the second dataset consisted only of cases where the sum score was at least 5. We then estimated three networks: one on the sample with all possible sum scores (hereafter, full sample network), one with our correction on the sample selected by a sum score criterion (hereafter, corrected network), and one with estimation as usual on the sample selected by a sum score criterion (hereafter, uncorrected network). The resulting networks are shown in Fig. 4.

If we were to find the results in Fig. 4 in an empirical network analysis, we could tentatively conclude a few things from a visual inspection of the three networks. First, the full sample network and the corrected network look almost exactly the same. Since there appears to be no differences between these two networks, we learn two things about the data-generating process: First, we learn that there is no evidence for a moderating effect of the sum score on the parameters of the Ising model: the parameters are the same for the whole sample regardless of the sum score. Moreover, it follows from this that there cannot be two or more different latent populations that are strongly related to the sum score and differ in terms of their network structure, i.e., the Ising model parameters. If we were to compare the uncorrected network with the full sample network, we might erroneously reach the opposite conclusions, since the networks look quite different: there is evidence for either a moderating effect of the sum score or the presence of different subpopulations. However, it is important to realize that differences between the uncorrected network and the full sample network are induced by conditioning on a common effect of the variables (selection bias or Berkson’s bias). Therefore, the uncorrected network cannot provide evidence for or against either a moderating effect of the sum score or the presence of different subpopulations in the data (similarly: Haslbeck et al., 2021b).

### An empirical case

To provide an example using empirical data, we used data from the National Comorbidity Screening Replication (NCS-R; Kessler & Merikangas, 2004). This dataset (*N* = 9,282) contains interview data on the presence or absence of the nine symptoms of a major depressive episode (MDE) and nine symptoms of generalized anxiety disorder (GAD) from the DSM-IV (American Psychiatric Association, 1994). For our example, we used only the MDE symptom data. The interviews had a skip structure, in which other symptoms were only assessed if either “depressed mood” or “loss of interest” was present and symptoms that were not assessed were recorded as absent (0’s) in the final dataset (see Kessler & Merikangas, 2004, for details). This dataset was previously analyzed by Borsboom and Cramer (2013) and by Forbes et al., (2017; see also Borsboom et al., 2017). Because of the skip structure, we decided to exclude the symptom of depressed mood from the empirical example, as the skip structure induces a negative association between depressed mood and loss of interest, which is not the result of merely a sum score criterion for the data as a whole, and hence our correction cannot account for it. Moreover, there were very few cases where this symptom was not present if any of the other symptoms were present (*N* = 37). This problem was less prominent for the symptom of loss of interest, where there were still 358 cases where the symptom was absent when any of the other symptoms were present (excluding depressed mood).

**Fig. 5:**
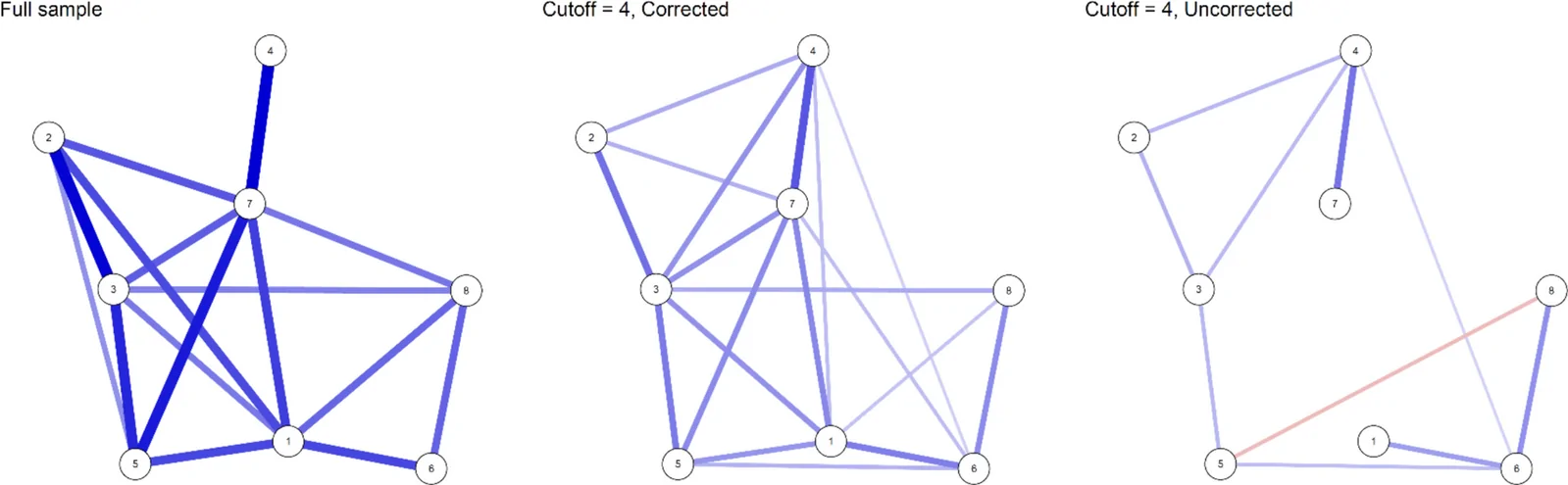
Three networks estimated from empirical data. *Note*. Three networks estimated from NSR-R data. All networks are estimated with a sample size of 2,052. The network on the left is estimated on a random sample without restrictions on the minimum sum score, while the networks in the middle and on the right are estimated on all cases in the data where the sum score reached at least 4. The middle network is estimated with our correction, and the right network without our correction. All networks were estimated with the *IsingSampler* package (α=0.05 with the AND rule)

**Fig. 6:**
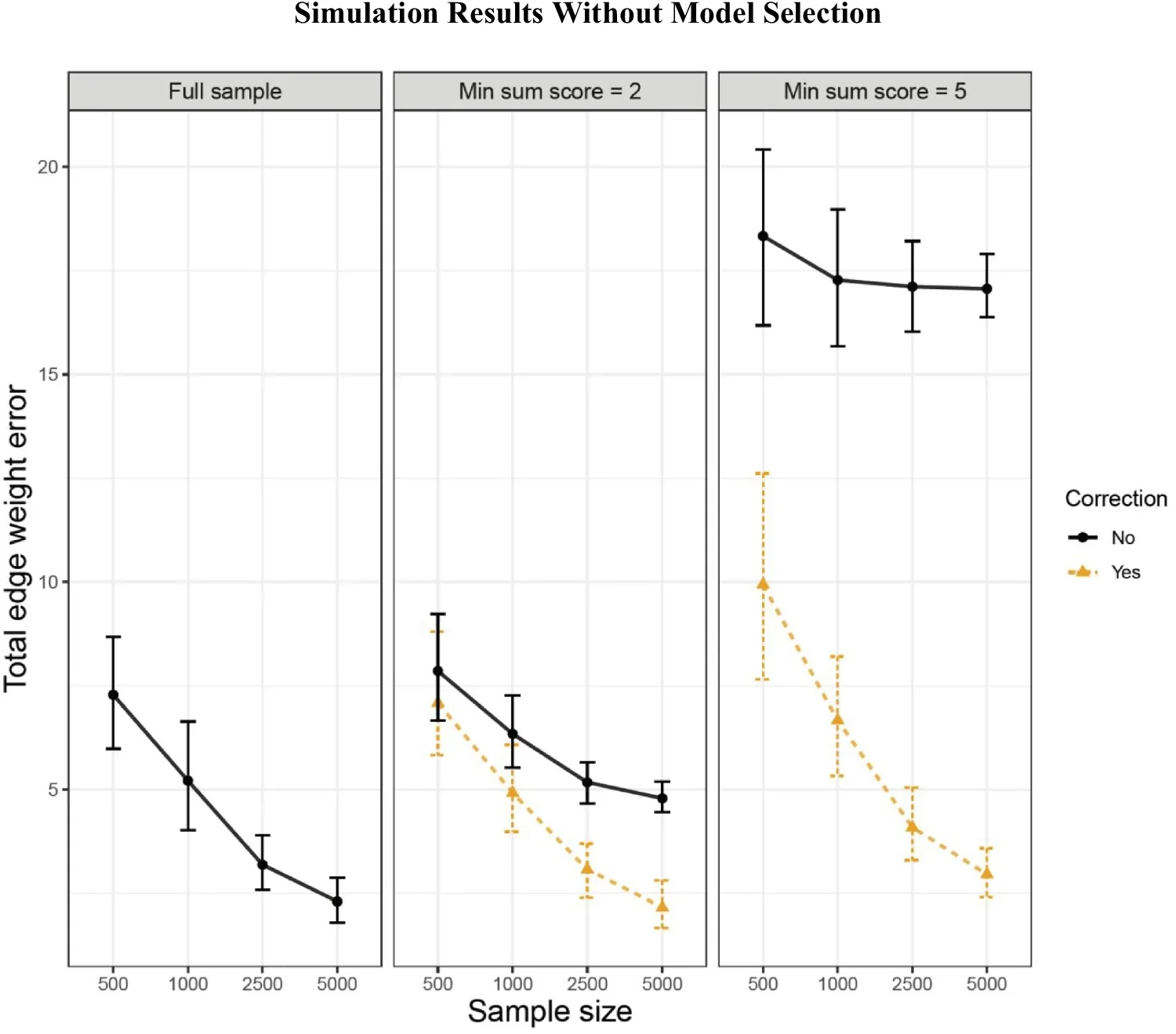
Simulation results without model selection. *Note.* The y-axis shows the mean total edge weight error of 100 simulations without model selection, and the x-axis shows the sample sizes of the simulated data. Due to strong overlap with multivariate estimation, we only show univariate estimation here. The golden dashed line shows our correction, and the black solid line shows estimation as usual. Note that we do not show all the outcome measures from the main text because they provide little information, as most edge weights are estimated as non-zero without model selection.

From the NSC-R data, we then again estimated three networks: one network using data with no restrictions on the sum score, one network using only data of people with a sum score of at least 4 symptoms with our correction, and one network of people with a sum score of at least 4 symptoms with estimation as usual. When estimating the network without restrictions on the sum score, we still used the same sample size as the other networks (*N* = 2,052) by randomly selecting cases, so that differences in sample size would not influence our results. The resulting networks are shown in Fig. 5.^5^

We note several differences between the full sample network and the corrected network: the average edge strength seems higher in the full sample than in the corrected networks. Moreover, there are edges missing in the corrected network that are present in the full sample network and the other way around. The edges that are missing in the corrected network could be missing because of reduced sensitivity. Our simulation study showed that a higher sum score cutoff still resulted in reduced sensitivity compared to estimation on the full sample, probably as a result of less variance in the variables after a sum score selection. However, it is unlikely that the edges in the full sample that are present in the corrected network are all false positives, as we use a fixed false positive rate of 0.01.

The observed differences suggest one of two mechanisms underlying the data. First, it could be that there is a single Ising model that produced the data, but the parameters of the Ising model are moderated by the sum score of symptoms. A model for which such a moderation could be observed would be an extension of the Ising model with interaction of an order higher than 2. The second option is that there are subpopulations in the data that have a different network structure, i.e., the Ising model, and are defined by a latent trait that closely aligns with the sum score. Our estimation method thus provides preliminary evidence that one of these two hypotheses is true but cannot distinguish between them. If a researcher thus had the hypothesis that there are two or more subpopulations with different Ising models in the data (e.g., a resilient and a vulnerable subpopulation), the conclusion they could draw is that the data are compatible with this hypothesis but not that the data support this hypothesis. As for the uncorrected network, this again merely shows that we pushed the conditional associations between the variables towards being negative, as the present edges are weaker than in the other networks, the network is sparser, and there is a negative edge present.

## Discussion

We introduced a way to correct for the bias resulting from a sum score selection in binary data modelled with the Ising model**.** We foresee applications of our corrections in two scenarios. In the first scenario, the target of inference is the full population, regardless of their sum score, but there is only data available on part of the population selected by a sum score cutoff criterion. The second scenario is when a researcher suspects the presence of two latent subpopulations that are described by different Ising models and that are imperfectly separated by a sum score cutoff criterion. Though one can never know if the second situation is actually true, we showed in an empirical example that our correction can be used to show that data is compatible with the presence of multiple latent populations that are closely related to the sum score. Importantly, even outside these two scenarios, networks estimated from data selected based on a sum score without correction should not be used to draw causal inferences, as the resulting associations are likely distorted by the selection procedure. Our simulation results demonstrate that the proposed correction method can effectively reduce selection bias after a sum score selection.

There are several aspects to consider when using sum score selection generally and the proposed correction specifically. A first consideration is that the correction is less effective if a smaller subset of the original data is used (i.e., if a higher cutoff value is chosen). This is immediate for the case in which the cutoff is the maximum possible sum score. However, also in less extreme cases, our simulation showed that a higher cutoff criterion leads to worse parameter estimates, especially in terms of sensitivity. This is likely caused by the fact that the model becomes more difficult to estimate with very low variance. While the problem of too little variance in a variable is not limited to situations where a sum score split is used, splitting on a sum score makes a lack of variance more likely because less data are used. Moreover, many mental disorders require for a diagnosis not only that a certain sum score of symptoms is reached but also that one of the key symptoms is present. For example, the DSM-5 requires either the symptom of depressed mood or loss of interest to be present for a diagnosis of major depressive disorder (MDD; American Psychiatry Association, 2013). Those symptoms will nearly always be present in cases from a diagnosed sample. One would likely need a very large sample size to achieve enough variance in these symptoms to estimate a network. If variance is absent or too low in a variable, it is impossible to fit a logistic regression with that variable as outcome, meaning a complete network model cannot be estimated. Ordinal data could be collected to avoid the problem of little variance data, as it has more variance naturally. For ordinal data, estimating a Gaussian graphical model (GGM; Lauritzen, 1996) is a popular approach for constructing a network (Epskamp & Fried, 2018), though this practice is not without its limitations (see Liddell & Kruschke, 2018). However, it is unclear whether the solution used in this study can be readily applied when estimating a GGM. Although the GGM has a known and tractable partition function, our correction relies on restricting the possible realizations when computing the normalizing constant, which is not straightforward to implement in the continuous GGM. Future studies could investigate ways to correct for selection bias when estimating the GGM or ordinal models as proposed in Marsman et al. (2025).

A second consideration is that if researchers use our correction to evaluate the hypothesis that there are multiple subpopulations related to the sum score that have different Ising models, the proposed method cannot provide evidence for this hypothesis, as discussed in Sect. 4. A more direct approach to answering this question would be to use latent class analysis (LCA; e.g., Weller et al., 2020) or for continuous data mixture modeling (e.g., McLachlan et al., 2019), as this allows researcher to directly test the existence of different subpopulations in the data. However, a major limitation of this type of analysis with respect to testing this hypothesis is that most software packages for fitting LCA variables are assumed to be independent within different classes, corresponding to empty Ising models. Therefore, traditional latent class analysis is thus not suitable for the situation in which the latent classes are defined by a different Ising model. Therefore, to directly evaluate the presence of subpopulations with different Ising models, we need a combination of LCA and network analysis, in which latent classes of different network models can be learned from the data, which has also been identified as an avenue for future research in Haslbeck et al. (2023)

Finally, we would like to emphasize that it remains vital that researchers think carefully about the reason why they want to do a sum score split. For example, it has often been suggested that the symptom networks of people with a disorder show more connectivity than people without a disorder (Van Borkulo et al., 2015; Cramer et al., 2016; Colli et al., 2024; whether this hypothesis will hold: Hoekstra et al., 2024). However, selecting data with a sum score cutoff criterion in a cross-sectional dataset as done in this study might not be an optimal way to investigate this. Cross-sectional networks show effects that exist between people in the sample and not processes within people in the sample (Borsboom et al., 2021; Hamaker et al., 2015). If the hypothesis is that strong connectivity between symptoms at the individual level poses a high risk for developing a mental order, this strong connectivity will not necessarily be found at the sample level. To study the vulnerability of a network, it thus seems more feasible to look at time-series network models, as they can reveal within-person processes (an overview of models is provided in Borsboom et al., 2021; Epskamp et al., 2018b). However, even when looking at within-person networks, it remains questionable whether differences in connectivity will be found with the current modeling techniques (for more details, see Hoekstra et al., 2024). Finally, if the sole reason to do network analysis after a sum score split is to see if there are qualitative differences between people with and without a mental disorder, this could also be tested with latent class analysis, if the assumed difference is not only in the network structure (Haslbeck et al., 2021b; Weller et al., 2020).

In summary, we introduced a method that effectively corrects for bias after a sum score selection and provide implementations in *IsingFit*, *IsingSampler*, *psychonetrics,* and *bootnet*. Because of these implementations, our method can easily be used by researchers. Since sum score selection is common in psychological research, especially when investigating the conditional associations between symptoms of a mental disorder, our method can be a valuable addition to this field.

## Data Availability

The R code and materials are available at https://github.com/Jesse291847/Correcting-for-selection-bias.

## Appendix A

### Aggregating symptoms from Cramer et al. (2016)

To aggregate the symptoms, we first simulated 400,000 cases from the disaggregated network. We then aggregated the symptoms in the simulated data, and estimated a network using *IsingFit* (Van Borkulo et al., 2016). However, the resulting network produced data that had almost no variance in the variables ‘depressed mood’ and ‘loss of interest’ after our chosen sum score selection. That is, there were almost no cases that reached the sum score with these symptoms absent, making it impossible to estimate a network model after a sum score selection. To remedy this, all edges between the ‘depressed mood’ and ‘loss of interest’ and any other symptoms were weakened. Moreover, we slightly increased the threshold parameter of other symptoms to make sure a larger proportion of simulated cases reached a higher sum score. This was achieved by multiplying the threshold parameters we estimated by .7.

**Table 1:** Symptom names and threshold parameters

Abbreviation	Symptom	Threshold
S1	Depressed mood	-1.73
S2	Loss of interest	-2.40
S3	Weight loss	-3.23
S4	Weight gain	-2.87
S5	Decreased appetite	-2.94
S6	Increased appetite	-2,93
S7	Insomnia	-2.27
S8	Hypersomnia	-3.34
S9	Psychomotor agitation	-2.38
S10	Psychomotor retardation	-3.25
S11	Fatigue	-2.12
S12	Feelings of worthlessness	-3.32
S13	Concentration problems	-3.03
S14	Thoughts of Death	-4.37

These are the threshold parameters from the true network shown in Figure 2 of the main text. Note that these are not empirical parameters, which is why the symptom names are not in the main text. See the work of Cramer et al., 2016 for the empirical parameters.

### A2 Variable names of empirical analysis section

**Table 2:** Variables in empirical analysis section

Variable number	Symptom
1	Loss of interest
2	Change in weight
3	Sleep problems
4	Psychomotor retardation/agitation
5	Fatigue
6	Self-reproach
7	Concentration Problems
8	Suicidal thought/behaviors

## Footnotes

1 From a causal inference perspective, the subsetting leads to confounding in the sense that the conditional distribution P(Y|X) is not the same as the P(Y|do(X)), where do(X) is the experimental manipulation of X (e.g., McElreath, 2020).

2 Some researchers have avoided this type of selection bias by using event-based selection methods, such as requiring exposure to traumatic events (e.g., experiencing spousal bereavement, as in Robinaugh et al., 2014, or an earthquake, as in McNally et al., 2015). This does not lead to attenuation of edges, as the selection criterion is a common cause of the symptoms (e.g., a trauma). While this reduces the risk of selection bias, such events are not always available, and may have limited generalizability to clinically defined populations. In contrast, when the selection criterion is a common effect of the symptoms, such as the sum score of the symptoms or help-seeking behavior, this can lead to attenuation of edges, rendering them unsuitable for generating causal hypotheses.

3 We note that the only the target population should be described by an Ising model for our correction to be effective. The other subpopulation(s) could also be captured by different models.

4 We use $\left[\mathrm{1,0}\right]$ encoding instead of the $\left[1,-1\right]$ encoding in our examples, as this is considered the most appropriate for symptom data (for details see Haslbeck et al., 2021a) and our correction is only implemented for this encoding.

5 We focus our analysis on the overall network structure; therefore, we do not show the variable names here. Interested readers can find them in Appendix A (Table 2).

## Code availability

The R code and materials are available at https://github.com/Jesse291847/Correcting-for-selection-bias.
